# Immune signature-based hepatocellular carcinoma subtypes may provide novel insights into therapy and prognosis predictions

**DOI:** 10.1186/s12935-021-02033-4

**Published:** 2021-06-30

**Authors:** Qiuxian Zheng, Qin Yang, Jiaming Zhou, Xinyu Gu, Haibo Zhou, Xuejun Dong, Haihong Zhu, Zhi Chen

**Affiliations:** 1 State Key Laboratory for Diagnosis and Treatment of Infectious Diseases, National Clinical Research Center for Infectious Diseases, Collaborative Innovation Center for Diagnosis and Treatment of Infectious Diseases, First Affiliated Hospital, School of Medicine, Zhejiang University, Hangzhou, 310003 Zhejiang China; 2 Department of Clinical Laboratory Center, Shaoxing People’s Hospital (Shaoxing Hospital, Zhejiang University School of Medicine), Shaoxing, 312000 China

**Keywords:** Immunotypes, Immune signature, Hepatocellular carcinoma, Tumour immune infiltration, Immunotherapy, Prognosis

## Abstract

**Background:**

Hepatocellular carcinoma (HCC) has a poor prognosis and has become the sixth most common malignancy worldwide due to its high incidence. Advanced approaches to therapy, including immunotherapeutic strategies, have played crucial roles in decreasing recurrence rates and improving clinical outcomes. The HCC microenvironment is important for both tumour carcinogenesis and immunogenicity, but a classification system based on immune signatures has not yet been comprehensively described.

**Methods:**

HCC datasets from The Cancer Genome Atlas (TCGA), the Gene Expression Omnibus (GEO), and the International Cancer Genome Consortium (ICGC) were used in this study. Gene set enrichment analysis (GSEA) and the ConsensusClusterPlus algorithm were used for clustering assessments. We scored immune cell infiltration and used linear discriminant analysis (LDA) to improve HCC classification accuracy. Pearson's correlation analyses were performed to assess relationships between immune signature indices and immunotherapies. In addition, weighted gene co-expression network analysis (WGCNA) was applied to identify candidate modules closely associated with immune signature indices.

**Results:**

Based on 152 immune signatures from HCC samples, we identified four distinct immune subtypes (IS1, IS2, IS3, and IS4). Subtypes IS1 and IS4 had more favourable prognoses than subtypes IS2 and IS3. These four subtypes also had different immune system characteristics. The IS1 subtype had the highest scores for IFNγ, cytolysis, angiogenesis, and immune cell infiltration among all subtypes. We also identified 11 potential genes, namely, TSPAN15, TSPO, METTL9, CD276, TP53I11, SPINT1, TSPO, TRABD2B, WARS2, C9ORF116, and LBH, that may represent potential immunological biomarkers for HCC. Furthermore, real-time PCR revealed that SPINT1, CD276, TSPO, TSPAN15, METTL9, and WARS2 expression was increased in HCC cells.

**Conclusions:**

The present gene-based immune signature classification and indexing may provide novel perspectives for both HCC immunotherapy management and prognosis prediction.

**Supplementary Information:**

The online version contains supplementary material available at 10.1186/s12935-021-02033-4.

## Background

Hepatocellular carcinoma (HCC) was the sixth most common type of malignancy and the fourth leading cause of cancer-related deaths in 2018 [1, 2]. Approximately 841,000 new cancer cases now occur per year, with more than 782,000 deaths [3]. The risk factors for HCC include viral infections (e.g., hepatitis B and C), alcohol consumption, obesity with non-alcohol fatty liver disease, and the high intake of aflatoxins [4]. Despite recent advances in HCC management, liver resection, transplantation, chemotherapy, radiotherapy, and molecular-targeting therapies that have improved HCC clinical outcomes to a certain degree [5], most patients are still diagnosed at advanced HCC stages and have limited therapeutic options [6–8]. Current curative rates are still poor for HCC because of its heterogeneity, high morbidity, high recurrence rate, metastases, and poor responsiveness to chemotherapy [9].

Immune checkpoint inhibitors (ICIs) have had promising, albeit limited, results as a type of HCC therapy [10]. Advances in single-cell RNA sequencing (RNA-seq) have provided novel landscape descriptions of the HCC immune system microenvironment [11]. Immune-related genes and tumour-infiltrating lymphocytes are known to play key roles in both carcinogenesis and tumour progression [12], and the cross-talk dynamics between infiltrating immune cells, immune cell cytokines, and tumour cells of the microenvironment govern hepatocarcinogenesis [13, 14]. A better understanding of the specific patterning of these dynamics may benefit immunotherapies, so a comprehensive approach to examining the diversity of the tumour-immune microenvironment is crucial for improving both responses to immunotherapy and prognosis predictions [15, 16]. In addition, immune cell infiltration has downstream functions in oncogenic pathways, and the microenvironment has a close relationship with responses to immunotherapies [17]. CD8+ T cells are known to be effective regulators of adaptive immunity for eliminating both pathogen-infected cells and tumour cells [18] and play an important role in tumour immunity [19]. M2-type macrophages are known to be crucial regulators in the tumour microenvironment through their inhibitory activity [20, 21]. Immune cells evolve with tumour progression, providing novel strategies to enhance the immunotherapy response.

Here, we evaluated immune cell signatures based on immune cell infiltration in HCC and identified four immune cell signature subtypes and their clinical outcomes. Among these subtypes, we found differences in the expression and distribution of classic chemotherapy-induced immune response markers and used immune cell scores to distinguish between them. Finally, we assessed immune gene expression profiles to comprehensively evaluate individual immune cell scores.

## Methods

### Databases

RNA-seq data from The Cancer Genome Atlas (TCGA, http://cancergenome.nih.gov), Gene Expression Omnibus (GEO, https://www.earthobservations.org), and International Cancer Genome Consortium (ICGC, https://www.icgc-argo.org) data portals were used for these analyses. Associated clinical information from these sites, including clinical outcomes, immune cell infiltrates, and responsiveness to immunotherapy, was also used.

### Data processing

Data from the GEO, TCGA, and ICGC were subjected to standardized data pre-processing and normalization. Only primary liver cancer samples were selected, and the first step was to remove samples with missing data. After filtering, we obtained 115 tumour tissue samples and 23,395 gene expression profiles from the GEO (GSE76427) and 369 primary liver cancer tissue samples and 25,342 gene expression profiles from the TCGA. We also pre-processed RNA-seq data from the ICGC LIRI-JP dataset (a total of 19,592 gene expression profiles), and the reads per kilobase of transcript per million mapped reads (RPKM) were converted into transcripts per million.

### Gene set enrichment analysis (GSEA)

**Fig. 1:**
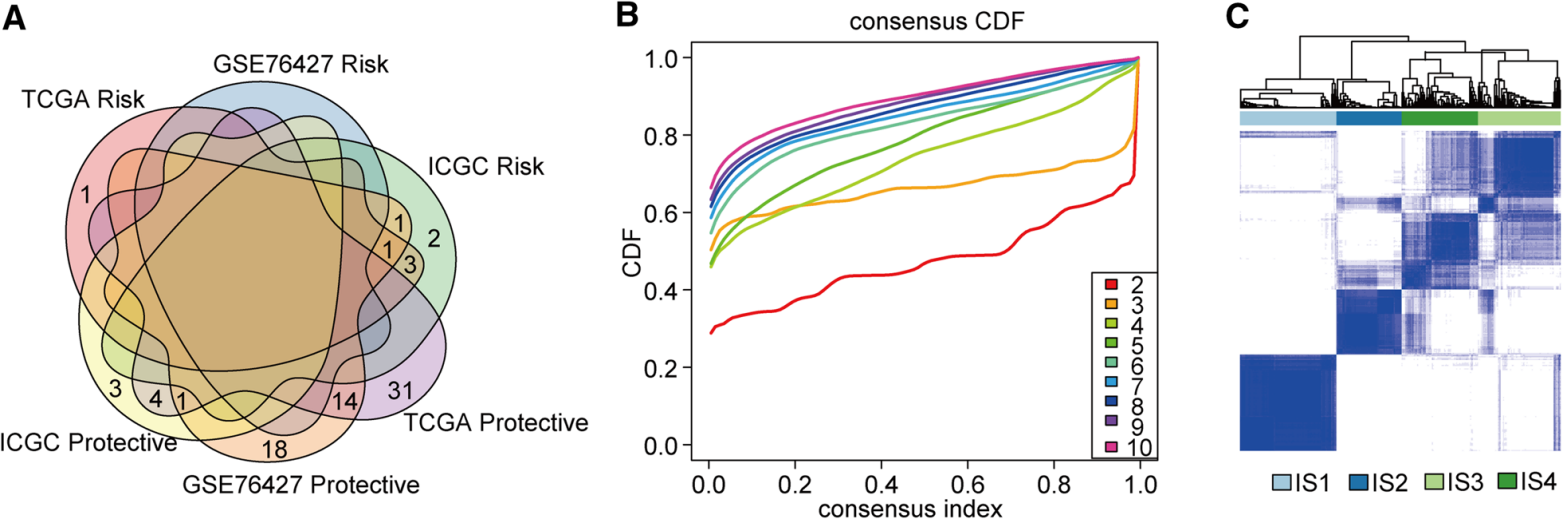
Identification of immunotypes in HCC patients. **a** A Venn diagram of the immune signatures showing the significant prognostic associations between the three cohorts les. **b** CDF curves of the TCGA cohort. **c** Heat map showing sample clustering results, with consensus k identified as 4

To determine differentially expressed RNAs, we performed GSEA using the limma package in R software (R-3.6.1.) and performed gene set variation analysis (GSVA) [22]. R software (version 1.24.0) was used to estimate the normalized enrichment scores (NESs) of the 152 immune signatures in the tumour microenvironment, as shown in Fig. 1a. These 152 immune signatures were collected from current and validated reports [23]. For a more in-depth analysis, we selected prognosis-related immune signatures from at least two cohorts. In total, 369 samples were assessed using the ConsensusClusterPlus tool in R software. The optimal cluster value was determined using the cumulative distribution function (CDF), and we identified four groups (Figs. 1a and Additional file 1: S1b) based on relative CDF delta area plot stability. These methods have been described previously [23, 24].

### Identification of immune-related subtypes and immune gene modules

Immune-related subtypes were identified using the ConsensusClusterPlus algorithm. A consensus matrix was first determined through consensus clustering to classify the samples [25]. For this process, the number of clusters was set between 2 and 10, and then consensus clustering was applied to classify the immune-related genes. The 152 NESs with their associated immune signatures were then used to classify the samples into different subtypes. The gene modules associated with drug resistance were also classified into different subgroups using this method. In-group proportions and Pearson's correlation analyses were then applied to validate the consistencies of these immune-related gene subtypes and modules. The specific methods have been described previously [24].

### Enrichment analysis

To further explore the biological functions of these gene modules, we conducted a single-sample GSEA to calculate the immune cell signature scores in 152 genes representing the HCC tumour microenvironment.

### Evaluation of immune subtypes and signatures

The log-rank test and both univariate and multivariate Cox regression methods were used to evaluate the prognostic values of the immune subtypes in both the training and sample sets, and analysis of variance was applied to assess both the immune subtypes and immune signatures.

### Immune landscape analysis

To comprehensively explore the immune landscape of the HCC samples, we applied a novel modelling technique with the ability to learn a set of embedding points in a low-dimensional space by retaining the inherent structure of high-dimensional data [26].

### Linear discriminant analysis (LDA)

To better quantify the distributions of immune characteristics for each subtype, we applied LDA to construct a categoricity index model. We used 23 prognosis-related resistance features and first performed a z-transformation on each segment. Fisher's LDA optimization standard stipulates that each group's centroid should be as dispersed as possible. We then determined a linear combination A and maximized the between-class variance in A relative to the within-class variance. The first two features of this model could clearly distinguish between different subtypes.

### Weighted gene co-expression network analysis (WGCNA) and cluster analysis

WGCNA was used to explore gene transcription information and to identify immune genes related to the co-expression modules [27]. Specifically, gene expression profiles were obtained from the TCGA database, the median absolute deviation was selected as > 50%, the cluster threshold was set at 9, and the β value was set at 9. Then, the expression matrix was transformed into a topology matrix. Average linkages were used in this analysis, with height = 0.25, deep split = 4, and min module size = 30 to obtain the modules.

### Cell culture

Human liver cancer HepG2 cells and normal human liver LO2 cells were cultured in complete (containing 4500 mg/L glucose, l-glutamine, sodium bicarbonate, no sodium pyruvate; liquid; suitable for cell culture) sterile-filtered Dulbecco's modified Eagle’s medium supplemented with penicillin and 10% foetal bovine serum (Gibco, NY, USA). The cells were grown in a humidified incubator with 5% CO_2_ at 37 °C.

### RNA extraction and reverse transcription quantitative PCR (RT-qPCR)

Total RNA was extracted from the cells using the RNeasy Plus Universal Mini Kit (50) (Qiagen, Hilden, Germany). To examine the mRNA expression levels of the 11 hub genes, RT-qPCR analysis was conducted with the PrimeScript™ RT Reagent Kit with gDNA Eraser (Perfect Real Time) according to the instructions provided in the kits. The primers are shown in Table 1. The following cycling conditions were applied: 95 °C for 5 min, followed by 40 cycles at 95 °C for 20 s and 60 °C for 30 s. GAPDH served as the internal control for normalization. The 2-ΔΔCT method was applied to calculate the mRNA expression levels of the 11 hub genes.

**Table 1:** The primers of 11 genes for RT-qPCR

Target	Sequence (5'–3')
TSPAN15 (F)	AAAGTTCAAGTGCTGTGGCG
TSPAN15 (R)	GCACACTGAAACGCTCCTTG
TSPO (F)	CTTTGGTGCCCGACAAATGG
TSPO (R)	CCGCCATACGCAGTAGTTGA
METTL9 (F)	TTGAGAAATCGGGCTGGCTAT
METTL9 (R)	AGTCTGTGGGTTTTCCAGTCT
CD276 (F)	GGGAGAAGGCTCCAAGACAG
CD276 (R)	GCCAGAGGGTAGGAGCTGTA
TP53I11 (F)	TGACCAGCTCTATGATGCGG
TP53I11 (R)	GTGAGCAGGGTCCATCGAAT
SPINT1 (F)	CTGGGCAGGCATAGACTTGA
SPINT1 (R)	TCTGGGTGGTCTGAGCTAGT
TRABD2B (F)	TCAAGCACTACAACTGCGGAGAC
TRABD2B (R)	TCCCCAGAAAGTGACCTGCTC
DYNC2LI1 (F)	GACGATGCCCAGTGAAACTC
DYNC2LI1 (R)	TCTTTTGGTGTGTTGTGCCC
WARS2 (F)	TGGGGAGTTCTTTCCAGTGC
WARS2 (R)	TCTGTTATTCGGACGGTGGC
C9ORF116 (F)	GAGAGGACCAGCGACTACTAC
C9ORF116 (R)	ACACGGAGACAGCCTTCTG
LBH (F)	TTGTGTCCACCTTGCCGAC
LBH (R)	CCTCAGTCATCTTGGCCGAT

### Statistical analysis

The PCR results were analysed with GraphPad Prism 7.0 software (GraphPad Software, Inc.). An unpaired Student's t-test was utilized to compare the differences between two groups. P < 0.05 was considered statistically significant.

## Results

### Identification of HCC sample subtypes based on immune signatures

To determine any co-relationships between HCC prognosis and enrichment scores based on immune signatures, we applied univariate survival analysis and identified closely associated immune signatures, 55 of which from the TCGA dataset (http://cancergenome.nih.gov), 35 of which from the GEO dataset (https://www.earthobservations.org), and 15 of which from the ICGC dataset (https://www.icgc-argo.org) were correlated with prognosis. There was only a small overlap between the three clusters, as illustrated in Fig. 1a, indicating considerable variability between individual immune signatures in the different datasets. The CDF was applied to categorize the optimal number of clusters. The cluster number identified as four showed relatively stable results (Fig. 1b). Immunotyping is of great importance for predicting the prognosis of and guiding immunotherapy for tumour patients. In this study, we used the ConsensusClusterPlus package in R software [25] to segregate the immune signatures of 369 samples from the TCGA into four subtypes: IS1, IS2, IS3, and IS4 (Fig. 1c). This kind of classification has been described by Huang et al. [2, 28] in studies of cholangiocarcinoma and pancreatic adenocarcinoma. These four subgroups have typical differences in immune characteristics.

### Prognostic analyses and evaluations of immune-associated genes in the four subtypes

**Fig. 2:**
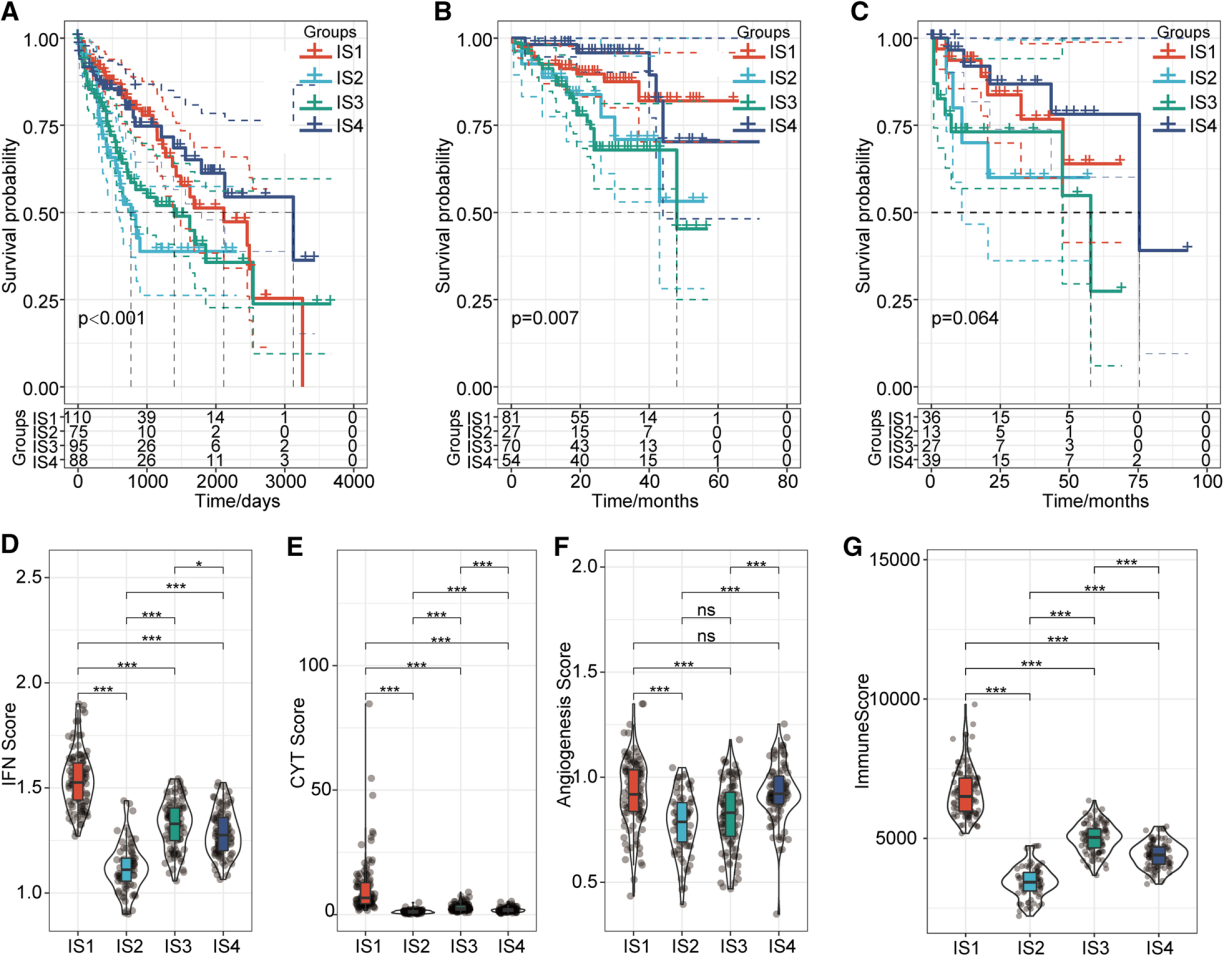
Overall survival analysis and immune-related score evaluations for the four subtypes. **a** Kaplan–Meier (KM) analysis of the four subtypes using the TCGA cohort. **b** KM curves for the four subtypes using the ICGC cohort. **c** KM curves for the four subtypes using the GEO cohort. **d** IFN scores of the four immunotypes. **e** CYT scores of the four immunotypes. **f** Angiogenesis scores of the four immunotypes. **g** Immune scores of the four immunotypes

Survival analyses of the four subgroups in the TCGA dataset revealed significant differences. Subgroups IS2 and IS3 had remarkably poorer prognoses than subgroups IS1 and IS4 (P < 0.001) (Fig. 2a). Consistent with these results, both the IS1 and IS4 subtypes of the ICGC cohort had much better prognoses than the IS2 and IS3 subtypes (P = 0.007) (Fig. 2b). Consistent with the results obtained from the ICGC and TCGA cohorts, the immune subtype in the GEO dataset showed that both the IS1 and IS4 subgroups had relatively better prognoses than the IS2 and IS3 subgroups (P = 0.064) (Fig. 2c). Interferon-gamma (IFNγ) is an important pro-inflammatory cytokine that functions in immune and inflammatory responses and in tumour immunosurveillance and homeostasis [29, 30]. Differences in the IFNγ scores also indicated considerable differences in immunotherapy tolerance [31]. We therefore calculated the IFNγ-related signature score for each sample. The results indicated remarkable differences between subtypes. The IS1 subtype had the highest IFNγ score, followed by the IS3, IS4, and IS2 subtypes (Fig. 2d). Cytolytic (CYT) activity within the local immune infiltrate has long been recognized as an anti-tumour immune response and has been recognized as a novel strategy USED to assess anticancer immunity [32, 33]. Specifically, the mRNA expression levels of both granzyme A (GZMA) and perforin (PRF1) have been reported to be novel indicators of CYT cancer immunity [34, 35]. The CYT scores for the four subgroups indicated significant differences between them, with subgroup IS1 having the highest CYT score, followed by subgroups IS3, IS4, and IS2 (Fig. 2e). In addition, genes related to angiogenesis have been reported to play essential roles in modulating the tumour microenvironment and the immune environment [36]. We calculated each sample's angiogenesis score and found that scores for the four groups were significantly different. The angiogenesis scores of the IS2 and IS3 subgroups were much lower than those of the IS1 and IS4 subgroups (Fig. 2f). Immune cell infiltration also plays an important role in the tumour microenvironment [34]. In the immune infiltration analysis, subgroup IS1 had the highest immune infiltration scores among all the subgroups, with score rankings of IS1 > IS3 > IS4 > IS2, and there were significant differences between subgroups (Fig. 2g). These results indicated that subgroups IS1 and IS4 had a better probability of survival than subgroups IS2 and IS3 in the TCGA, ICGC and GEO cohorts. Subgroup IS1 had relatively higher IFNγ, CYT, angiogenesis, and immune infiltration scores than the other subgroups. In addition, higher IFNγ, CYT, angiogenesis, and immune infiltration scores suggest a better prognosis. These results suggest that the immunotypes of HCC patients are reproducible and stable. Overall, the immunotype has the potential to become a valuable prognostic biomarker of and effective immunotherapy evaluation indicator in HCC.

### Subtype differences in the expression of genes related to immune responses, immune cell infiltration, and immune checkpoints

**Fig. 3:**
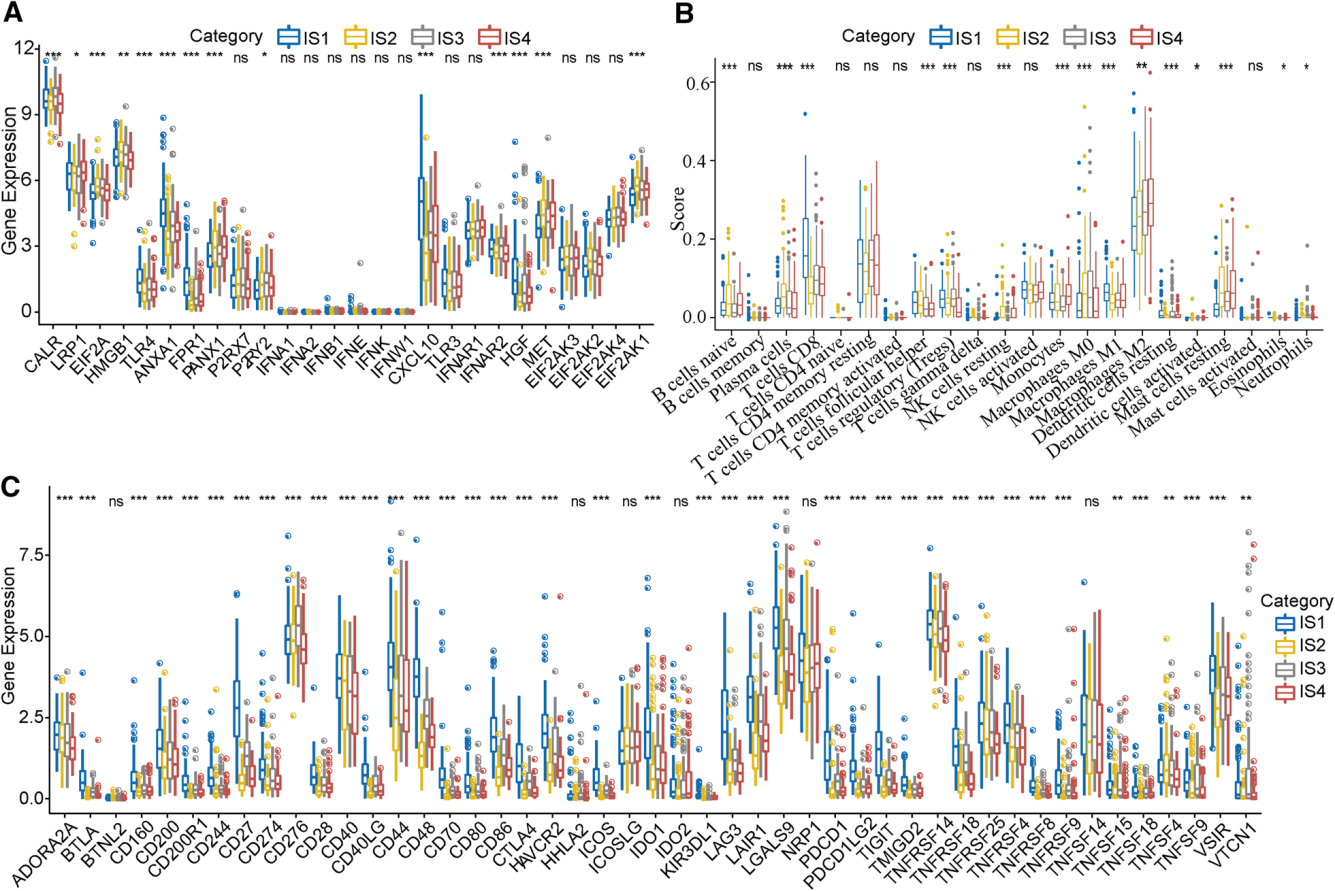
Immune infiltration gene scores in the four subtypes. **a** Differences in the expression and distribution of classic chemotherapy-induced immune response genes in the TCGA dataset. **b** Differences in the expression and distribution of immune cell-associated genes in the TCGA dataset. **c** Differences in the expression and distribution of immune checkpoint genes in the TCGA dataset

We selected 26 immune response marker genes because of their relatively high expression levels and examined their differential expression. Fourteen of these (53.8%) showed significant differential expression in the four immune-related subtypes: CALR, LRP1, EIF2A, HMGB1, TLR4, ANXA1, FPR1, PANX1, CXCL10, IFNAR2, HGF, MET, and EIF2AK1 (P < 0.001). A statistically significant difference in LRP1 and P2RY2 expression was noted between subgroups. In addition, the expression of the immune response markers TLR4, ANXA1, FPR1, CXCL10, and HGF was significantly higher in the IS1 subgroup than in the IS2, IS3 and IS4 subgroups (Fig. 3a). Next, we determined the immune scores for the four subgroups using the 22 immune cell values and determined that the infiltration scores among the subgroups were remarkably different, especially concerning naïve B cells, plasma cells, CD8+ T cells, follicular helper T cells, Tregs, resting NK cells, monocytes, M0 macrophages, M1 macrophages, M2 macrophages, resting dendritic cells, activated dendritic cells, and resting mast cells. The IS1 subgroup had the highest scores for CD8+ T cells, follicular T cells, and M1 macrophages; the IS1 subgroup also had the lowest scores for M0 macrophages, M2 macrophages, and resting mast cells. Consistent with the previous results, the IS2 subgroup had the lowest score of CD8+ T cells. The IS4 subgroup had a high ratio of infiltrating M2 macrophages. Among the many differences observed between the four subtypes, the IS2 subgroup had high ratios of infiltrating plasma cells, B cells, and naïve B cells (Fig. 3b). Immunotherapies have shown promising therapeutic efficacies for a variety of tumours, and ICI therapies have significantly transformed treatments for solid tumours [37–39]. We determined the expression levels of immune checkpoint genes in the four subgroups and found that 41 of 47 genes (87%) were significantly differentially expressed. With the exceptions of BTNL2, HHLA2, ICOSLG, IDO2, NRP1, and TNFRSF14, all the other immune checkpoint genes, namely, ADORA2A, BTLA, CD160, CD200, CD200R1, CD244, CD27, CD274, CD276, CD28, CD40, CD40LG, CD44, CD48, CD70, CD80, CD86, CTLA4, HAVCR2, ICOS, IDO1, KIR3DL1, LAG3, LAIR1, LGALS9, PDCD1, PDCD1LG2, TIGIT, TMIGD2, TNFRSF14, TNFRSF18, TNFRSF25, TNFRSF4, TNFRSF8, TNFRSF9, TNFSF15, TNFSF18, TNFSF4, TNFSF9, VISIR, and VTCN1, were remarkably differentially expressed among the four subtypes (Fig. 3c). In summary, the immunotype has a close relationship with immune-associated cells and modulators. These results have demonstrated that the differential expression of genes related to immune responses, immune cell infiltration, and immune checkpoints is associated with HCC prognosis, and this analysis may provide novel therapeutic targets and prognostic predictors for HCC.

### Subgroup analyses of immune cell infiltration ratios, oncogenesis pathways, and interactions with other pan-cancer immune subtypes

**Fig. 4:**
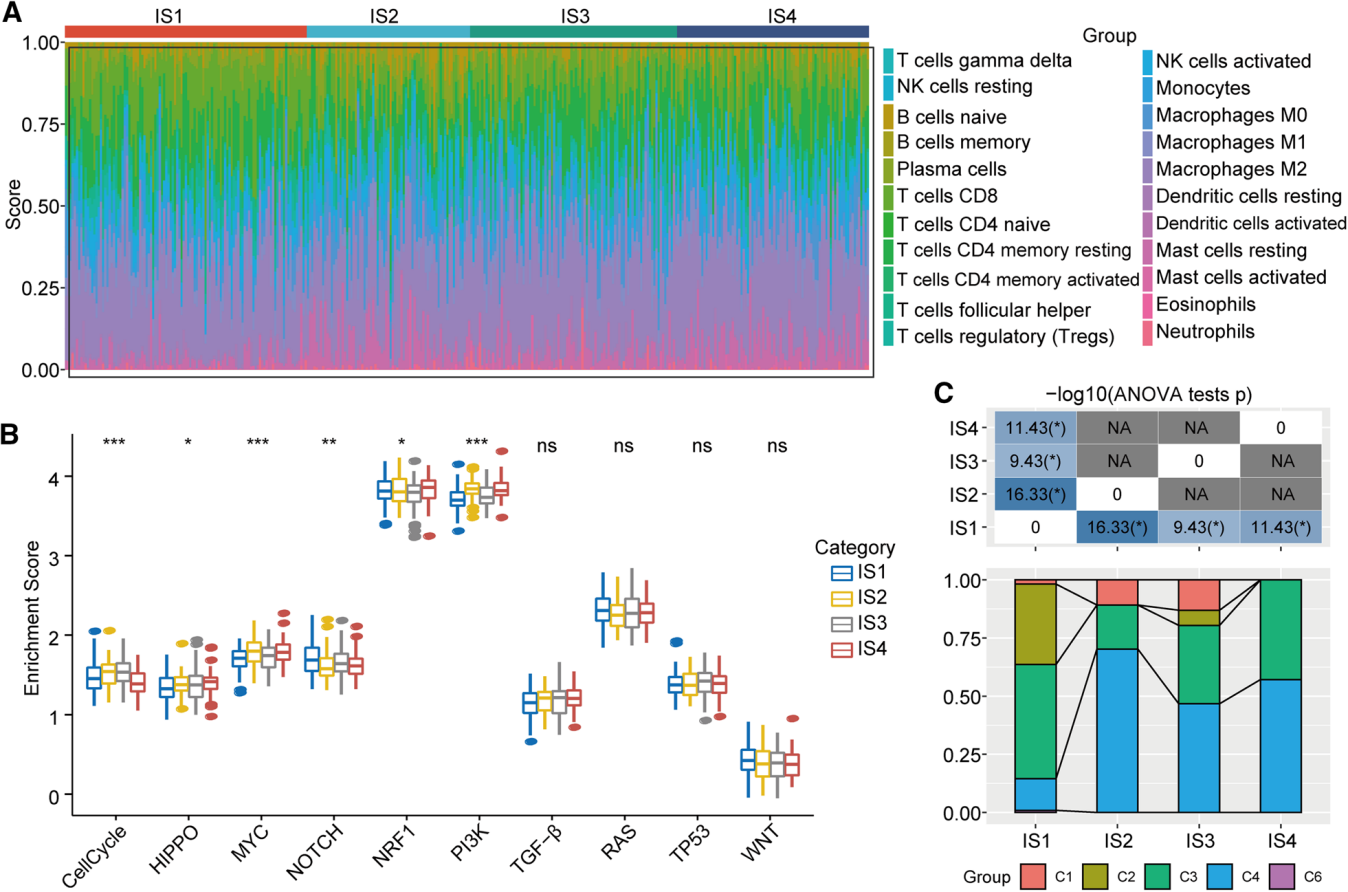
Distributions of infiltrating immune cells and pathway enrichment analysis. **a** Proportions of 22 immune cell types in the different subgroups. **b** Differences in the enrichment scores of 10 tumour pathways in the different subgroups. **c** Intersections between the four immune molecular subtypes described herein and six previously reported pan-cancer immune molecular subtypes

To further investigate immune cell infiltration ratios among the four immunotypes, we investigated whether the immune cell infiltration composition ratios were significantly different. For example, in the IS1 subtype, the results indicated that T cells and monocytes accounted for the majority of the infiltrating immune cells, whereas the IS2 and IS3 subgroups had a relatively low ratio of T cells (Fig. 4a). Furthermore, we performed functional enrichment analysis, and the results showed that the cell cycle signalling pathway, MYC signalling pathway, and PI3K signalling pathway, followed by the NOTCH pathway, HIPPO pathway, and NRF1 pathway, were significantly different between the four immunotypes. The IS1 subgroup had comparatively low enrichment scores in the cell cycle pathway, MYC pathway, and PI3K pathway. The subgroup associated with the poorest outcome, IS2, had high enrichment scores in the cell cycle, HIPPO, MYC, NRF1, and PI3K signalling pathways (Fig. 4b). Therefore, we validated that lower oncogenic enrichment scores might indicate a better prognosis. To better understand the relationship between the intersection of HCC immunotypes and six pan-cancer immunotypes, we extracted molecular subtype data from a previous study [38] and determined that the IS1 subtype was composed mainly of the C2 and C3 subtypes, the IS2 subtype was composed mainly of the C4 subtype, and the IS3 and IS4 subtypes were composed mainly of the C3 and C4 subtypes (Fig. 4c). The results from our study are similar to those from previous studies to some extent. In addition, these results suggest that the four immunotypes described herein could be used to supplement HCC-associated immune classification.

### Immune feature quantification in the four subtypes

**Fig. 5:**
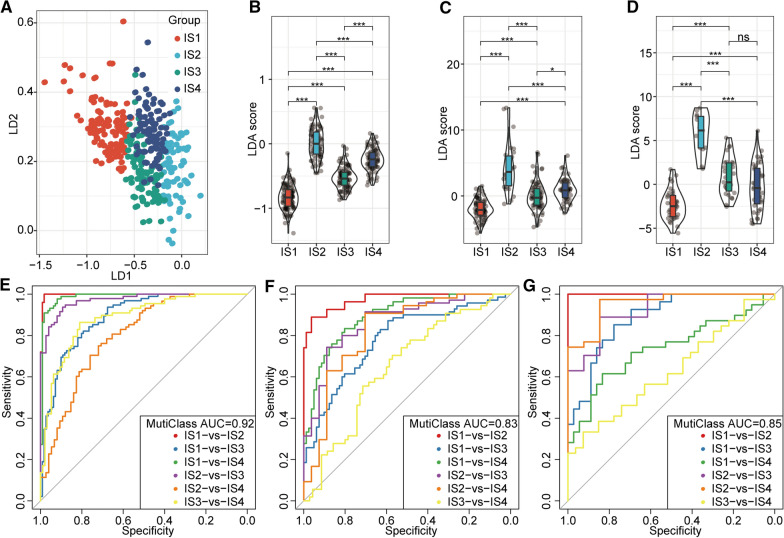
Evaluations of immune characteristic indices. **a** Relationships between the first two immune characteristic subtype indices (LD1 and LD2) and the four immune subtypes. **b** Differences in immune characteristic indices among the different subtypes in the TCGA dataset. **c** Differences in immune characteristic indices among the different subtypes in the ICGC dataset. **d** Differences in immune characteristic indices among the different subtypes in the GEO dataset. **e** ROC curves for the immune characteristic indices in the TCGA dataset. **f** ROC curves for the immune characteristic indices in the ICGC dataset. **g** ROC curves for the immune characteristic indices in the GEO dataset

We applied the LDA model to validate data centrally. Different colours represent different immunotypes, and the results showed that their distribution was concentrated, and the distance between the categories was obvious (Fig. 5a). Furthermore, the calculated LDA scores for each of the four TCGA subgroups showed marked differences: the IS2 subgroup had the highest LDA score, and the IS1 subgroup had the lowest LDA score (Fig. 5b). Consistent with these results, the subgroup LDA scores for the ICGC and GEO databases were also significantly different; the IS2 LDA scores were much higher, and the IS1 scores were lower (Fig. 5c and d). The LDA scores from 3 different databases indicated a high degree of consistency. These results suggest that our immunotype has good stability in different databases. We also applied receiver operating characteristic (ROC) curves to assess the classification performance of feature indices in the TCGA dataset. The area-under-the-curve (AUC) value was 0.92 (Fig. 5e) in the TCGA dataset. Likewise, the multiclass AUC value was 0.83 for the ICGC dataset (Fig. 5f) and 0.85 for the GEO dataset (Fig. 5g). These multiclass AUCs verified that the immunotyping model has good classification efficiency.

### Assessments of LDA scores and immunotherapy responses

**Fig. 6:**
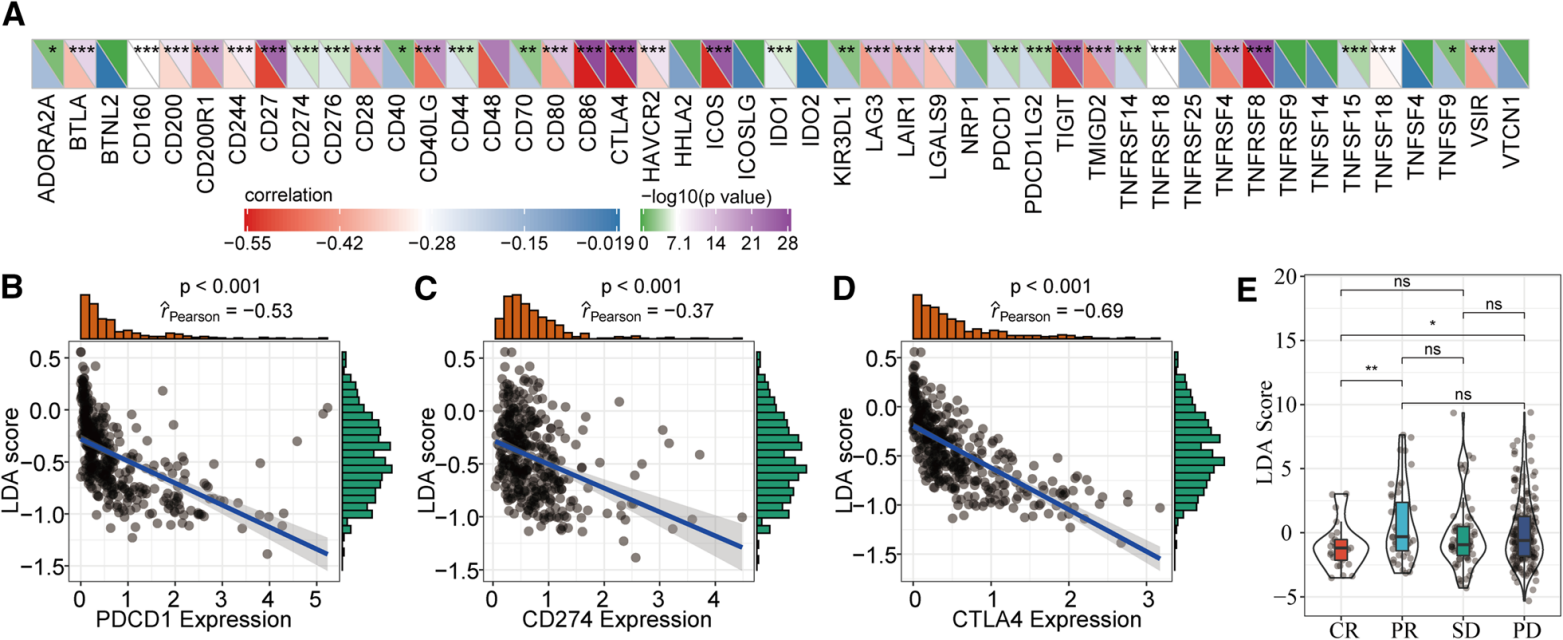
Assessments of immune signature indices. **a** Relationship between immune characteristic indices and expression of immune checkpoint genes. **b** Correlations between immune characteristic indices and PDCD1 gene expression. **c** Correlations between immune characteristic indices and CD274 gene expression. **d** Correlations between immune characteristic indices and CTLA4 gene expression. **e** Differences between immune characteristic indices and treatment response states

We calculated the correlations (Pearson's coefficients) between the immune characteristic indices and the expression of 47 immune checkpoint genes. These correlations between LDA scores and immune checkpoint gene expression are illustrated in Fig. 6a. The expression of most immune checkpoint genes, especially CD27, CD86, CTLA4, ICOS, TIGIT, and TNFRF8, was negatively correlated with the immune signatures (Fig. 6a). We further investigated the most thoroughly studied immune checkpoint molecules. The results indicated that PDCD1 was significantly negatively associated with the LDA score (P < 0.001, R =  − 0.053) (Fig. 6b), CD274 expression was markedly negatively associated with the LDA score (P < 0.001, R =  − 0.37) (Fig. 6c), and CTLA4 expression was remarkably negatively correlated with the LDA score (P < 0.001, R =  − 0.69) (Fig. 6d). These results showed that the immune characteristic indices and LDA score were significantly negatively correlated with most immune checkpoint molecules. In addition, we obtained a dataset of gene expression profiles from a previous study of patients with metastatic urothelial cancer who were treated with PD-L1 and calculated both their immune characteristic indices and their responses to different immunotherapies. There were significant differences between the four patient subgroups (static disease, SD; progressive disease, PD; partial response, PR; and complete response, CR) in immune characteristic index values (Fig. 6e). These differences were observed to be related to CR/PR and to CR/PD. We also calculated the immune indices of our samples and obtained three datasets (GSE18728, GSE5462, and GSE20181) related to tumour chemical therapies to explore any correlations between the immune signatures and chemotherapies. The results indicated no significant differences in chemotherapy responses among the four subgroups (Additional file 2: Fig. S2a–c). Notably, in the GSE20181 dataset, there were no significant differences between the immune signature indices when the chemotherapy response group was compared to the non-response group (Additional file 2: Fig. S2d). However, compared to the non-treatment group, the immune signature indices were markedly decreased in the chemotherapy treatment group. Furthermore, with extended treatment time, the immune signature indices gradually decreased (Additional file 2: Fig. S2e).

### Co-expressed genes related to immune signatures

**Fig. 7:**
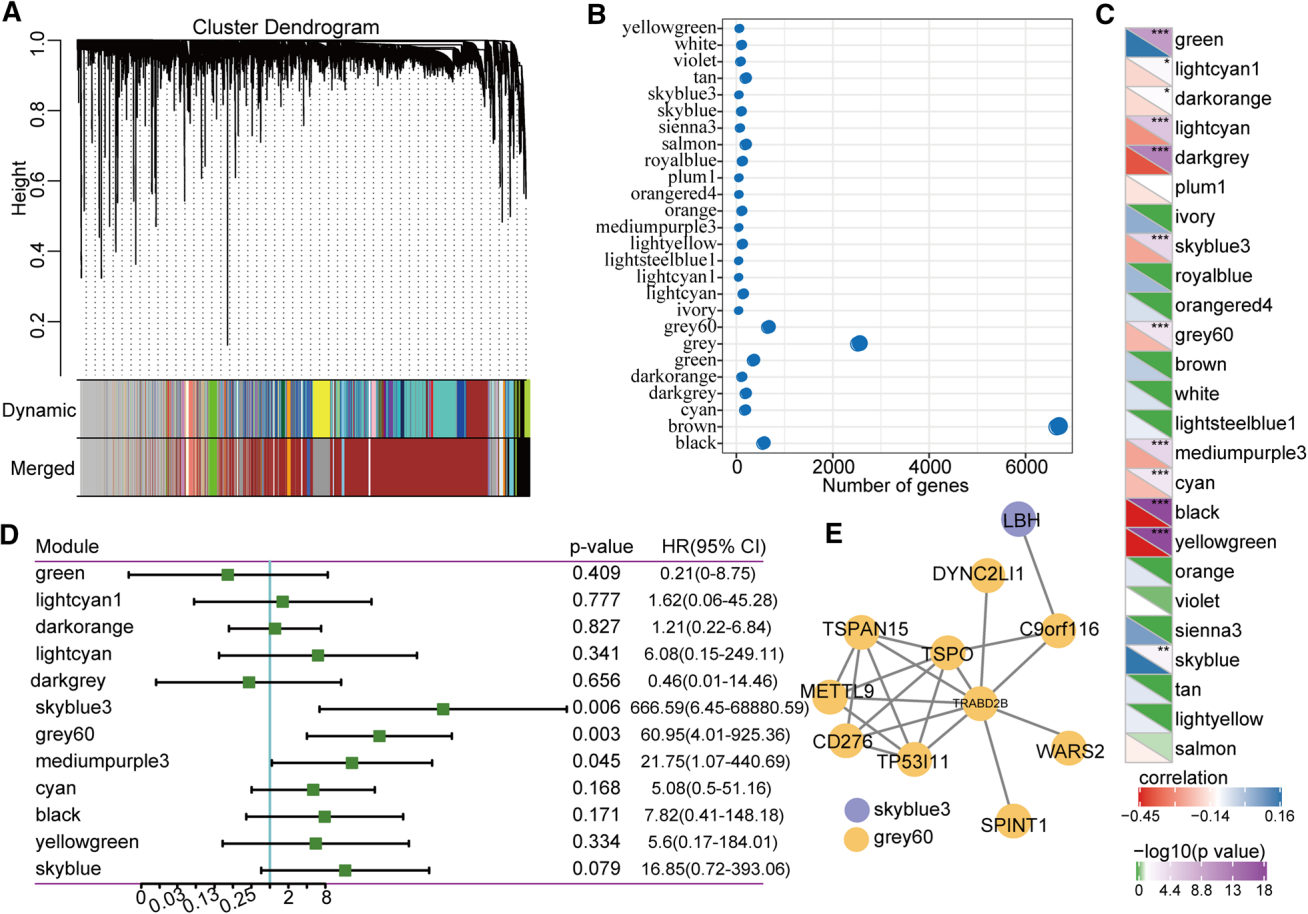
Identification of immune gene co-expression modules. **a** Cluster dendrogram of all differentially expressed genes/lncRNAs based on a dissimilarity measure (1-TOM). **b** The number of genes found in each module. **c** The correlations between modules and immune characteristic indices. **d.**Prognostic correlations between the modules and immune characteristic indices. **e** Network analysis of potential gene markers related to immune characteristic indices

**Fig. 8:**
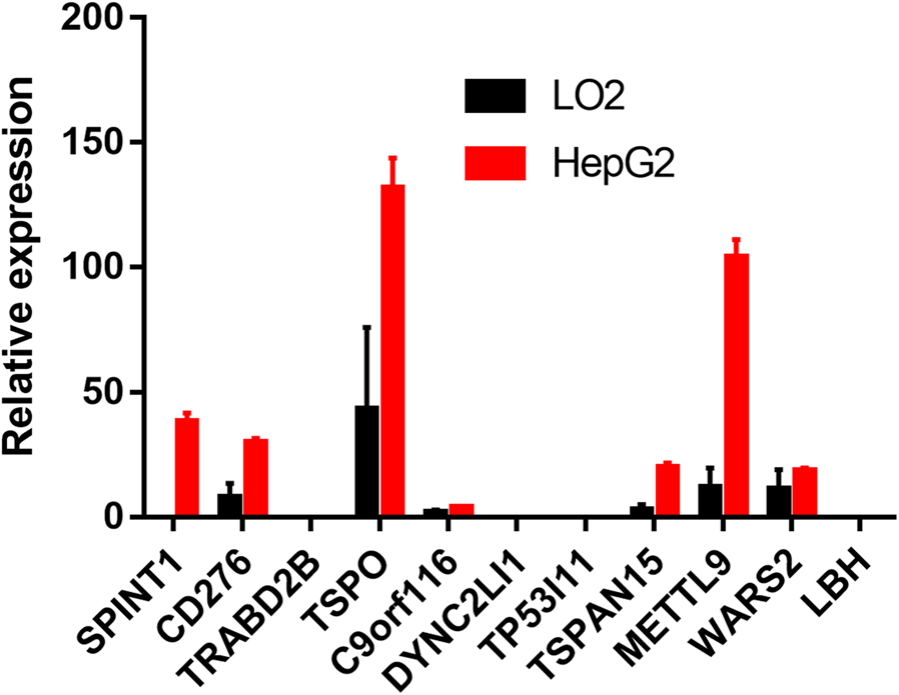
The mRNA expression levels of 11 hub genes in Hep-G2 and LO2 cells

The HCC samples were clustered based on their gene expression profiles using distributed-cluster analysis. To ensure that the network was scale-free, we chose a soft threshold of β = 9. Next, we converted the representation matrix into an adjacency and then transferred the matrix into a topological matrix. The average-linkage hierarchy clustering method was applied to cluster genes. We also set the minimum number of genes in each gene network module as 30 in accordance with the standard of the hybrid dynamic shear tree. The dynamic shearing method was used to determine the gene modules, and then the eigengene value of each module was calculated. Then, cluster analysis was performed on the modules, and the modules close to each other were merged into new modules with the following parameters: height = 0.25, deep split = 4, and min module size = 30. A total of 26 modules were obtained (Fig. 7a), and the transcripts within these modules were distributed, as shown in Fig. 7b. In addition, we investigated any co-relationships between these module features and immune signatures (Fig. 7c). Of the 26 modules, 12 had significant overlaps with differentially expressed genes. We also investigated any co-relationships between immune signature indices and module-based prognoses and observed that sky blue 3, grey 60, and medium purple 3 all indicated significant differences in prognosis (Fig. 7d). We also identified 11 prognosis-associated genes that were co-expressed, 10 of which originated from the grey 60 module (Fig. 7e). These 11 genes were TSPAN15, TSPO, METTL9, CD276, TP53I11, SPINT1, TRABD2B, WARS2, C9ORF116, and LBH. We performed RT-qPCR to validate their expression levels in Hep-G2 and LO2 cells. The results showed that most of these immune signature-based genes, such as SPINT1, CD276, TSPO, TSPAN15, METTL9, and WARS2, were significantly upregulated in HCC cell lines (Fig. 8). Collectively, these findings demonstrate that these hub genes have the potential to become novel immunological biomarkers of HCC.

## Discussion

HCC is a leading cause of cancer-related mortality, with most patients in advanced stages of the disease when they are initially diagnosed [40]. These advanced-stage patients, with high rates of recurrence and metastases, are no longer good candidates for surgical resection [40, 41]. HCC carcinogenesis and progression are complex, with interactions between a variety of genetic backgrounds and tumour microenvironments. Immunotherapies, especially ICIs, have become promising immunotherapeutic strategies for HCC [42]. Due to surgical resection limitations, other combinatorial options with chemotherapeutics and immunotherapies have gained increasing attention for advanced HCC treatments [43]. However, immunotherapy response rates remain both low and heterogeneous [41]. The identification of novel biomarkers and construction of an immune-based classification scheme for HCC may provide new approaches to improve responses to immunotherapies and overcome drug resistance.

Immunogenomics has provided evidence to genetically characterize immune cell and cancer cell interactions. Tumour-node-metastasis stage, tumour grade, and microvascular invasion are the most common parameters used for HCC assessments, especially for a differential diagnosis, treatment selection, and prognosis prediction [44]. However, these assessments cannot be used to evaluate a patient’s immune status and therefore cannot guide the selection of HCC immunotherapies.

The HCC immune microenvironment is characterized by both intratumoural and intertumoural heterogeneity [45]. The recognition of HCC immune signatures based on immunotherapy-related genes has provided a great shift in the effects of immunotherapy, and further refinement of these signature-based classifications may facilitate more sensitive immunotherapies for the different subtypes identified. Here, we identified four HCC immune subtypes based on 152 immune signature genes, and these subtypes exhibited distinct differences in patient prognoses. Several studies have classified HCC patients into different subgroups based on genomic profiles in tumour tissues and adjacent normal tissues. Li et al. [46] identified five gene expression subtypes based on immune profiles of HCC patients. Julien Calderaro et al. [47] estimated six molecular phenotypes based on genomic mutations of HCC. Bidkhori et al. [48] also developed three different metabolic and signalling pathways associated with cancer types based on HCC tumour tissues. In our previous study, we also identified four subtypes based on glycolytic and cholesterogenic genes in HCC [49]. Gong et al. [50] applied paired tumour and adjacent nontumour tissues from GEO and discovered three clinically relevant subtypes based on immune features and hallmark genes and nontumour samples in HCC. Our work is mainly focused on the immune signatures of HCC tumour tissues, and our analysis adopted three classical HCC datasets from TCGA, GEO and ICGC and identified four distinct immune subtypes. Each of these studies have its’ own unique advantages and have potential supplement with each other. Thus, discussing HCC immune subtypes from different perspectives might achieve better immune signature classifications and provide a comprehensive understanding of HCC immunotherapies.

In addition, we validated these results using other independent datasets, and the subtypes also exhibited remarkably different immune characteristics and responses to immunotherapy and chemotherapy. This classification based on immune signature genes provides a more comprehensive understanding of both the immune microenvironment and the management of HCC. Previous studies have also suggested classifications based on immune-related genes. Zhang et al. [51] classified three HCC subtypes (immunocompetent, immunodeficient, and immunosuppressive), and Sia et al. [34] identified two immune gene-based subclasses based on adaptive or exhausted immune responses. In addition, Kurebayashi et al. [52] assessed the HCC immune microenvironment and determined three distinct immune subtypes (immune-high, immune-mid, and immune-low) based on intratumoural heterogeneity. However, these studies did not reflect the comprehensive immune status of HCC. Our classification scheme provides novel perspectives for immunotherapies, oncolytic viruses, anti-angiogenic agents, and radiotherapy. It may therefore improve the current HCC classification for better disease management.

HCC is a complex disease that is combined with an immune-tolerant microenvironment [34]. We proposed four immunotypes, IS1, IS2, IS3, and IS4, of which HCC patients had significantly different immune-associated characteristics. Patients in the IS1 and IS4 subgroups had better prognoses than those in the IS2 and IS3 subgroups. We constructed an immune signature-based classification for HCC prognosis prediction that can also provide more efficient strategies for immunotherapies. The present immunophenotypic classification of the four subtypes also involved investigations into common mutations, chemotherapeutically induced immune responses, immune features, and pathway characteristics. In addition, our analysis was carried out based on transcriptome information from different databases, which makes it more clinically feasible for the clinical evaluation of HCC and decision making. In addition, we identified eleven immune signatures, namely, TSPAN15, TSPO, METTL9, CD276, TP53I11, SPINT1, TRABD2B, WARS2, C9ORF116, DYNC2LI1 and LBH, which may serve as potential HCC biomarkers, and the use of such signature-based indices may shed light on novel targets for both personalized treatments and immunotherapies for HCC patients. Furthermore, we applied PCR analysis and validated that most of these immune signature-based genes, such as SPINT1, CD276, TSPO, TSPAN15, METTL9, and WARS2, were significantly upregulated in HCC cell lines. These studies indicate that these immune signature-based genes have a close relationship with cancer progression and immune infiltration, but further explorations are still needed. However, validation of such experiments will require additional comprehensive and comparative research to confirm the efficiency of this classification for clinical evaluations and decision making. The immunotypes and immune characteristics of HCC might also be suitable for other cancers. However, there are limitations to our analysis. First, the eleven immune signature genes need further validation in the clinic, in vivo and in vitro. Second, transcriptome information was obtained from liver tissues after surgery and could not be accurately predicted prior to starting HCC; therefore, a better circulatory biomarker released from tumour cells and tumour-associated immune cells into the blood is urgently needed. Third, further functional and underlying mechanistic investigations and validation of the eleven immune-associated hub genes in HCC are needed.

## Conclusions

In conclusion, we first identified four immunotypes in HCC. These subgroups also showed differential responses to immunotherapy and chemotherapy. We investigated 11 genes, namely, TSPAN15, TSPO, METTL9, CD276, TP53I11, SPINT1, TRABD2B, SPINT, WARS2, C9ORF116, and LBH, which might act as immunotherapy targets for HCC. We also validated that most of these genes were significantly upregulated in cancer cells. We propose a practical HCC immune-associated classification and identify immune signature-associated hub genes that may improve HCC immunotherapy management and prognosis predictions.

## Supplementary Information

**Additional file 1: Fig. S1.** Clustering analysis to identify four subtypes of HCC. A. The CDF Delta area curve of TCGA cohort samples. B. Clustering tree of each sample. C. Analysis of the scale-free fit index for various soft-thresholding powers (β). D. Analysis of the mean connectivity for various soft-thresholding powers

**Additional file 2: Fig. S2.** Identification and validation of difference subtypes. A. There is no significant difference in chemotherapy response in different subtypes of the TCGA dataset. B. There is no significant difference in chemotherapy response in different subtypes of the ICGC dataset. C. There is no significant difference in chemotherapy response in different subtypes of the GEO dataset. D. The difference in immune characteristic index between chemotherapy response and non-response group in GSE20181 dataset. E. Differences in immune characteristic index between different chemotherapy treatment time and non-treatment group in the GSE20181 dataset.

## Data Availability

The authors declare that the data supporting the findings of this study are available within the article.

## Abbreviations

HCC: Hepatocellular carcinoma
TCGA: The Cancer Genome Atlas
GEO: Gene Expression Omnibus GEO
ICGC: International Cancer Genome Consortium
GSEA: Gene set enrichment analysis
LDA: Linear discriminant analysis
WGCNA: Weighted gene co-expression network analysis
IS: Immune subtype
ICIs: Immune checkpoint inhibitors
RNA-seq: RNA sequencing
CDF: Cumulative distribution function
RPKM: Reads per kilobase of transcript per million mapped reads
GSEA: Gene set enrichment analysis
RT-q PCR: *Reverse* Transcription quantitative PCR
IFNγ: Interferon-gamma
CYT: GZMA: Cytolytic; granzyme
PRF1: Perforin
ROC: Receiver operating characteristic
SD: Static disease
PD: Progressive disease
PR: Partial response
CR: Complete response
