# Supplementary figures and images for: Immune signature-based hepatocellular carcinoma subtypes may provide novel insights into therapy and prognosis predictions
Source: Cancer Cell Int. 2021 Jun 30;21:330. doi: 10.1186/s12935-021-02033-4 (PMC8243542; doi:10.1186/s12935-021-02033-4)

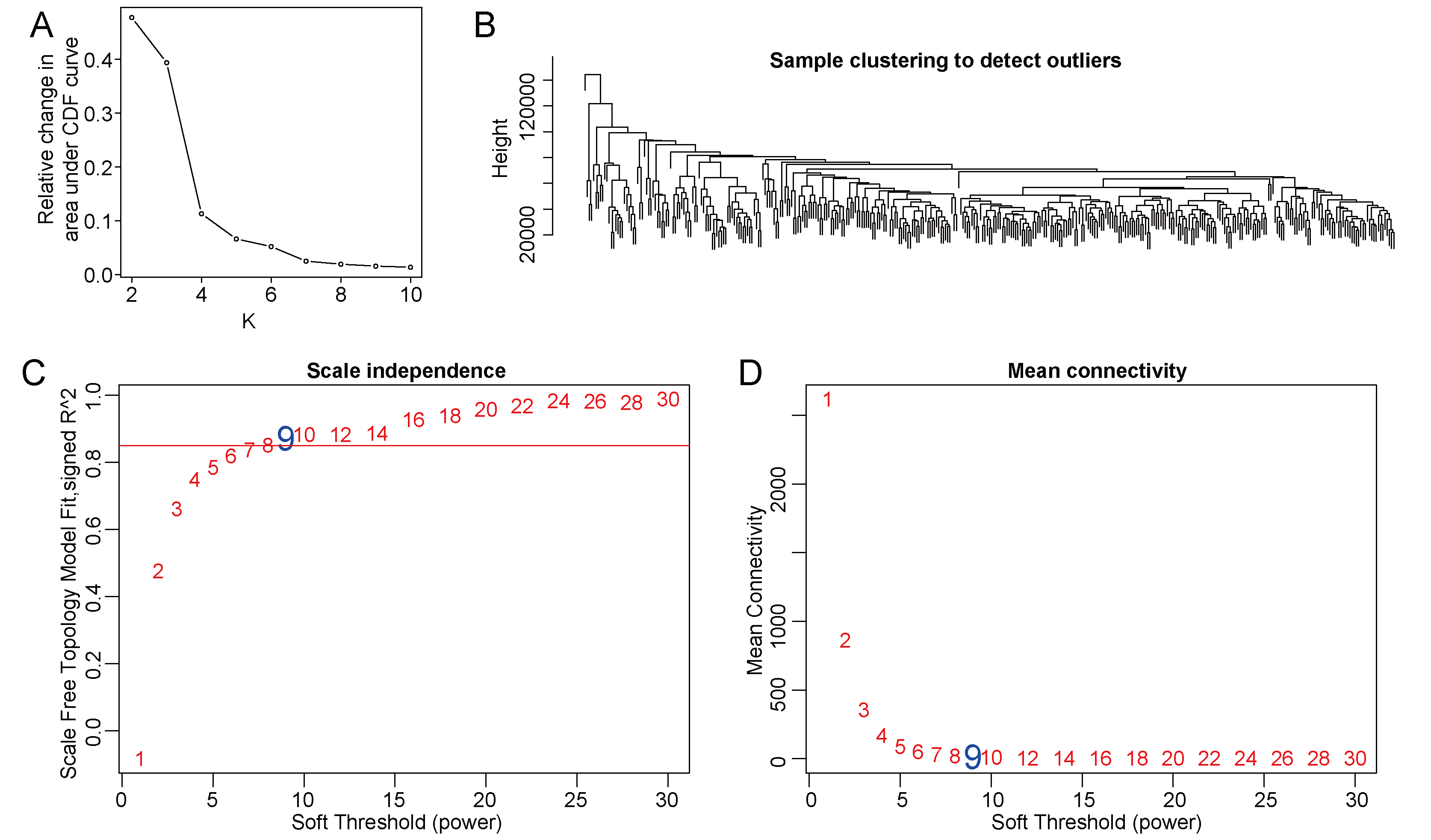

Supplement: Supplementary file 1 — Additional file 1: Fig. S1. Clustering analysis to identify four subtypes of HCC. A. The CDF Delta area curve of TCGA cohort samples. B. Clustering tree of each sample. C. Analysis of the scale-free fit index for various soft-thresholding powers (β). D. Analysis of the mean connectivity for various soft-thresholding powers [file 12935_2021_2033_MOESM1_ESM.tif]

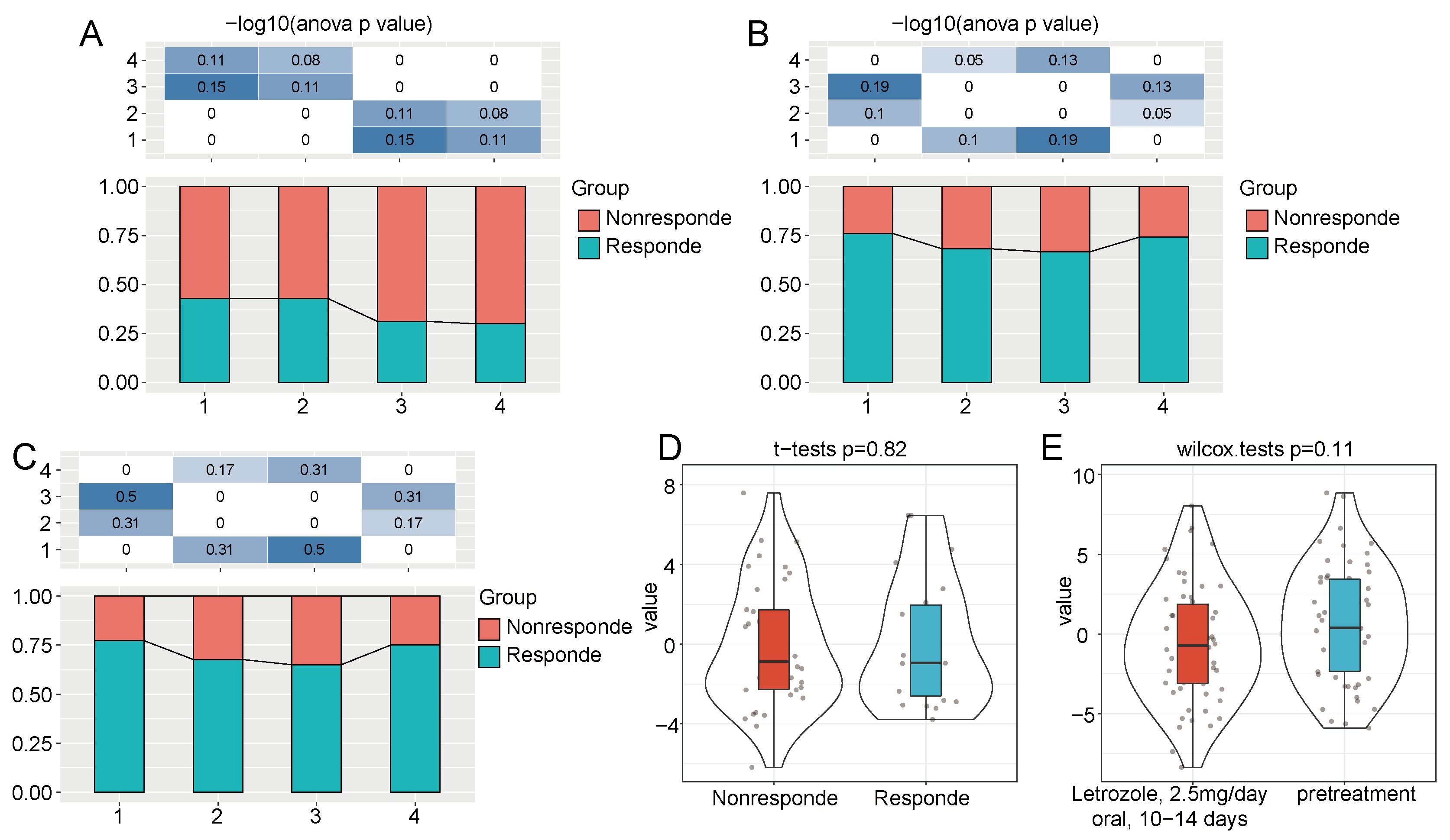

Supplement: Supplementary file 2 — Additional file 2: Fig. S2. Identification and validation of difference subtypes. A. There is no significant difference in chemotherapy response in different subtypes of the TCGA dataset. B. There is no significant difference in chemotherapy response in different subtypes of the ICGC dataset. C. There is no significant difference in chemotherapy response in different subtypes of the GEO dataset. D. The difference in immune characteristic index between chemotherapy response and non-response group in GSE20181 dataset. E. Differences in immune characteristic index between different chemotherapy treatment time and non-treatment group in the GSE20181 dataset. [file 12935_2021_2033_MOESM2_ESM.tif]
